# LC-MS/MS Method Validation for Quantification of Nirmatrelvir in Human Plasma

**DOI:** 10.1155/ianc/6625833

**Published:** 2025-11-17

**Authors:** Natpapat Kaewkhao, Joel Tarning, Daniel Blessborn

**Affiliations:** ^1^Mahidol Oxford Tropical Medicine Research Unit, Faculty of Tropical Medicine, Mahidol University, Bangkok, Thailand; ^2^Nuffield Department of Clinical Medicine, Centre for Tropical Medicine & Global Health, University of Oxford, Oxford, UK; ^3^Infectious Diseases Data Observatory (IDDO), University of Oxford, Oxford, UK

**Keywords:** COVID-19, LC-MS/MS, nirmatrelvir, plasma, validation

## Abstract

Nirmatrelvir, a key antiviral agent in the treatment of COVID-19, requires accurate and reliable monitoring of drug levels to optimize therapeutic efficacy. In this study, we developed and validated a sensitive and specific LC-MS/MS method for the quantification of nirmatrelvir in human plasma. The method includes nirmatrelvir-D9 as an internal standard, with quantification achieved using selected reaction monitoring in positive electrospray ionization mode, targeting m/z 500.3 ⟶ 110.1 for nirmatrelvir and m/z 509.3 ⟶ 110.1 for nirmatrelvir-D9. Sample preparation involved a simple phospholipid removal step using 96-well plate and automated liquid handler, which improved efficiency in a high-throughput process. The validated method, following international bioanalytical guidelines, demonstrated a linear range from 10.9 to 3013 ng/mL. Intra- and interassay precisions were both below 15%. All validation tests meet the criteria for matrix effect, carryover, dilution integrity, and stability. The method offers a rapid analysis time of 2 min per sample and provides highly accurate, reproducible results, making it a valuable tool for evaluating the pharmacokinetics of nirmatrelvir in clinical settings.

**Trial Registration:**

ClinicalTrials.gov identifier: NCT05041907

## 1. Introduction

The COVID-19 pandemic, which began in late 2019, has underscored global health challenges and the urgent need to strengthen resilience against health emergencies [1, 2]. Various drugs, including remdesivir, monoclonal antibodies, and baricitinib [3], as well as vaccines [4], have been evaluated for safety and efficacy. Among these advancements, nirmatrelvir, a key component of the oral antiviral Paxlovid, has gained importance for targeting the main protease of SARS-CoV-2. Accurate plasma quantification is critical to support clinical pharmacokinetics and optimize therapeutic efficacy [5], highlighting the need for validated bioanalytical methods that meet regulatory standards for effective clinical application.

Liquid chromatography-tandem mass spectrometry (LC-MS/MS) is the gold-standard analytical technique for quantifying drugs in biological matrices due to its high sensitivity, specificity, and reproducibility. Several published methods for measuring nirmatrelvir in plasma sample rely on mass spectrometry (Table 1). These validated methods, with LLOQs of 2–50 ng/mL, are suitable for clinical use, offering short analysis times and employing a simple, rapid process of protein precipitation [6–11]. Additionally, the use of 50 μL [7, 9], 100 μL [6, 8, 10], and 200 μL [11] of plasma per sample makes these methods efficient for clinical analysis. However, there are still rooms for improvements of current methods in order to develop a novel assay that combines an ultrashort analysis time, high sensitivity, low sample volume, robust performance, and the implementation of the assay in a high-throughput laboratory setting, making it ideal for large-scale clinical studies.

## 2. Materials and Methods

### 2.1. Ethical Conduct of Research

Ethical approval for collecting human volunteer blood in this study was granted by the Ethics Committee of the Faculty of Tropical Medicine, Mahidol University (MUTM 2020-068-04) in October 2023. Data collection began the same month. Informed consent was obtained from participants who signed forms and underwent screening, including vital signs and routine lab tests. Blood samples were anonymized at the Healthy Volunteer Ward before being sent to the clinical laboratory.

### 2.2. Chemicals and Reagents

The reference standards of nirmatrelvir (purity 98.3%) were obtained from MedChemExpress (Monmouth Junction, NJ, USA). Its isotope-labeled internal standard nirmatrelvir-D9 (purity 92.7%) was acquired from Clearsynth (Mumbai, India). Purified water was produced using a Direct-Q 5 UV water system (Ultrapure water, UPW18.2 MΩ·cm; Merck, Germany). MS acetonitrile was obtained from JT Baker (Phillipsburg, NJ, USA). Ammonium formate and formic acid were purchased from Honeywell (formerly Fluka) (Charlotte, NC, USA). HPLC methanol was acquired from Fisher Chemical (Pittsburgh, PA, USA). Dimethyl sulfoxide (DMSO) molecular biology grade was obtained from Sigma-Aldrich (St. Louis, MO, USA).

### 2.3. Standards and Working Solutions, Linearity, and Quality Controls

Stock solutions (1 mg/mL) of nirmatrelvir and its isotope-labeled internal standard nirmatrelvir-D9 were prepared in DMSO, and working solutions were diluted in acetonitrile:water (50:50, v/v). The final concentration of organic solvents in plasma samples was kept below 5% for all preparations.

The linear range was 10.9–3013 ng/mL. Four QC levels, QC1 (LLOQ × 3), QC2 (low), QC3 (midrange), and QC4 (close to the highest standard), were used to assess accuracy and precision. They were prepared at 32.7, 450, 1522, and 2440 ng/mL. The lowest and highest concentrations in the calibration range represent the lower limit of quantification (LLOQ) and upper limit of quantification (ULOQ), respectively, and overcurve samples prepared at about 2 × ULOQ and diluted tenfold with blank plasma before analyzed were also assessed for accuracy and precision. The spiked plasma sample was stored at −80°C until analysis.

### 2.4. Sample Preparation

An automated liquid handler platform (Freedom Evo100 platform, TECAN, Mannedorf, Switzerland) was used for the sample preparation process. Nirmatrelvir spiked plasma (50 μL) was precipitated with 750 μL acetonitrile containing 300 ng/mL nirmatrelvir-D9. The plate was mixed using a Mixmate (600 rpm, 20 min) and centrifuged (1100 × g, 2 min). The supernatant (250 μL) was transferred to an Ostro 96-well plate (Waters Corporation, MA, USA). A vacuum of 0.5 inch Hg was applied, increasing by 0.5 inch Hg every 30 s until all wells were dry, not exceeding 10-inch Hg to avoid possible cross-contamination. The collected sample eluate was diluted with 700 μL acetonitrile:water (50:50, v/v).

### 2.5. LC-MS/MS

The Dionex Ultimate 3000 LC system (Thermo Fisher Scientific, MA, USA) included a binary pump, vacuum degasser, and temperature-controlled autosampler and column compartment. Separation was achieved using a 50 mm × 2.0 mm I.D., 5 μm Gemini C18 column (Phenomenex, Torrance, CA, USA) with a C18 precolumn (4 mm × 2 mm I.D., 3.0 μm). The mobile phase was acetonitrile and 10 mM ammonium formate with 0.5% formic acid (60:40, v/v) at a flow rate of 0.4 mL/minute under isocratic conditions. Injection volume was 2 μL, with an ultrashort total runtime of 2 min per sample.

Nirmatrelvir quantification was achieved via selected reaction monitoring (SRM), with transitions m/z 500.3 ⟶ 110.1 for quantitation and m/z 500.3 ⟶ 319.3 as qualifier. The isotope-labeled internal standard, nirmatrelvir-D9, was quantified using the transitions m/z 509.3 ⟶ 110.1 and m/z 509.3 ⟶ 328.3 as qualifier. Analysis was performed on an API 5000 mass spectrometer (Sciex, MA, USA) with a TurboV ionization source in positive ion mode. Ion spray voltage was 5500 V, and the drying gas temperature was 500°C. Data were processed using Analyst 1.7.2 (Sciex, MA, USA).

### 2.6. Method Validation

Method validation followed FDA (2018) [12] and ICH M10 (2022) [13] guidelines, assessing selectivity, specificity, linearity, dilution integrity, precision, accuracy, recovery, and stability using spiked EDTA plasma samples (nonhemolyzed and hemolyzed). Overcurve samples were diluted 10-fold with blank plasma and validated for dilution integrity, enabling reliable quantification of clinical samples above the ULOQ. Accuracy was measured as mean relative error (%) and precision as coefficient of variation (%CV), with ANOVA used to calculate the precision of the method (intra- and interassay variability). Process efficiency was defined as the peak response of each QC sample relative to the average peak of a spiked reference solution and absolute recovery as the peak response relative to a postspiked sample. The matrix effect was determined by comparing the postspiked sample signal response to that of the spiked reference solution.

### 2.7. Clinical Application

The applicability of the validated method was demonstrated in the PLATCOV trial. The trial was conducted according to Good Clinical Practice principles. In Thailand, the trial was approved by the Faculty of Tropical Medicine Ethics Committee, Mahidol University (reference TMEC 21-058) and by the Oxford University Tropical Research Ethics Committee (Oxford, UK; reference 24-21). The developed method was used to analyze a total of 458 patient plasma samples, supporting the pharmacokinetic and therapeutic evaluation of nirmatrelvir. Additionally, incurred sample reanalysis (ISR) was performed, confirming reproducibility between the initial and subsequent analyses. ISR criteria were met, with more than 67% of reanalyzed samples showing less than ±20% difference from the initial analysis, demonstrating the method's reliability and robustness [14].

## 3. Result and Discussion

### 3.1. Method Validation

The validation tests for selectivity, specificity, precision, accuracy, recovery, and stability were conducted using both nonhemolyzed and hemolyzed spiked EDTA plasma samples, while linearity and dilution integrity were evaluated with nonhaemolyzed spiked EDTA plasma.

**Figure 1:**
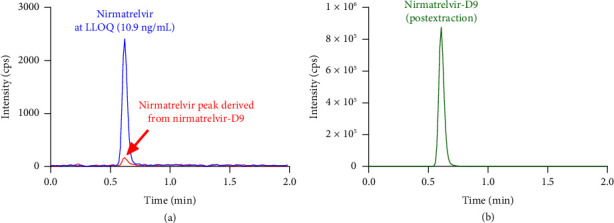
Extracted ion chromatograms of (a) spiked plasma with nirmatrelvir at the LLOQ overlaid with the interfering peak derived from a blank plasma sample extracted with isotope-labeled internal standard nirmatrelvir-D9 (300 ng/mL). (b) The original nirmatrelvir-D9 signal intensity in the blank plasma sample extracted with 300 ng/mL of internal standard.

Selectivity and specificity of the method were demonstrated by evaluating blank EDTA plasma samples from six different donors, including hemolyzed EDTA plasma. There was no significant interference observed (<20% of the LLOQ) at the retention time of nirmatrelvir (Supporting Information Figure S1). Additionally, no interference was detected after injecting ritonavir (50 ng/mL) or paracetamol (100 ng/mL) as neat solutions (Supporting Information Figure S1). No carryover of nirmatrelvir or nirmatrelvir-D9 was detected when injecting blank solution following five ULOQ injections (Supporting Information Figure S2). The absence of potential interference between the analyte and its internal standard at ULOQ plasma level suggests that nirmatrelvir reference standard purity 98.3% was high enough and that there were no significant cross-contamination in mass spectrometric signals between nirmatrelvir (m/z 500.3 ⟶ 110.1) and nirmatrelvir-D9 (m/z 509.3 ⟶ 110.1) (Supporting Information Figure S3). However, a minor cross-contamination from nirmatrelvir-D9 (purity of 92.67%) to nirmatrelvir was observed when extracted plasma was injected (postextraction concentration ∼35 ng/mL) (Supporting Information Figure S4), but the contribution was less than 9% of the LLOQ signal of nirmatrelvir and well within the acceptable criterion of <20% of the LLOQ (Figure 1). This difference in interference levels can be attributed to the purity of the compounds.

Before analysis, system suitability was assessed by injecting a minimum of five replicates of the QC solution 43.8 ng/mL. A relative standard deviation of the area responses of less than 3% was needed to ensure adequate LC-MS system performance. Nirmatrelvir standard calibration range was set between 10.9 and 3013 ng/mL, and ULOQ was limited by the signal intensity observed in the LC-MS detector. Higher concentrations risked saturating the mass spectrometer, requiring a larger extraction solvent volume to dilute the sample and maintain detector linearity at ULOQ [15, 16]. Saturation can lead to inaccurate quantification, as the measured spectrum no longer reflects the true concentration of the analyte [15]. The linear range of the developed method should still be able to capture the maximum concentrations seen with standard dosing in COVID-19 [9, 17, 18]. Standard curves were constructed at 10.9, 54.3, 209, 628, 1883, and 3013 ng/mL, using duplicates at each concentration where blank plasma samples, both with and without the isotope-labeled internal standard nirmatrelvir-D9, were analyzed alongside the standards. LLOQ and ULOQ were defined as the lowest and highest calibration points. All back-calculated mean concentrations of the calibration standards were within ±5% of their nominal values (Supporting Information Table S1). Back-calculated concentrations were used to assess linearity and verify the regression model. Both linear and quadratic regression models were evaluated, with the 1/*X*^2^ weighted linear regression model provided the best fit to the collected data [19]. This weighted linear regression showed minimal bias, as indicated by the lowest sum of relative residuals (equation (1)) and a high correlation between signal intensity and nominal concentrations (*r* > 0.99). The 1/*X*^2^ weighted quadratic regression model had a slightly higher sum of relative residuals, suggesting a less optimal fit. The linear model was prioritized not only due to its superior performance but also because it is the simplest model, making it easier to interpret and generalize. Nonweighted regression was also tested but showed very poor predictive performance.

(1) $$\begin{array}{c} \text{Relative residual}=\frac{\left|\text{predicted conc}.-\text{nominal conc}.\right|}{\text{nomical conc}.}. \end{array}$$

**Figure 2:**
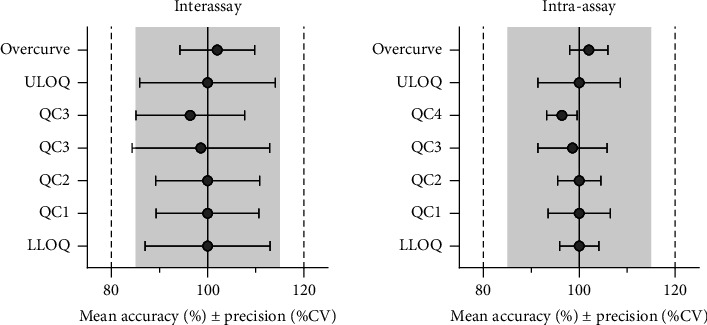
Accuracy and precision of QCs, LLOQ, ULOQ, and overcurve in EDTA plasma samples (*n* = 5). Circles represent mean accuracy, with their associated inter- and intra-assay precision (ANOVA).

Dilution integrity was tested by analyzing five replicates across four batch runs, using samples containing 6623 ng/mL of nirmatrelvir, diluted 10-fold with blank EDTA plasma. Intra-assay and interassay precision and accuracy for diluted nirmatrelvir samples met the acceptance criteria (Figure 2 and Supporting Table S2). This validated procedure ensures reliable quantification of samples exceeding the ULOQ.

Accuracy and precision were assessed in both nonhemolyzed and hemolyzed EDTA plasma samples using LLOQ, ULOQ and four QC levels. Five replicates of each sample were quantified over 4 days. Accuracy and precision for nirmatrelvir met the acceptance criteria. Intra-assay and interassay precisions (calculated using a single factor ANOVA) were all below 15% for all QC levels (Figure 2 and Supporting Information Table S2). Additionally, accuracy and precision for hemolyzed samples at QC1 and QC4 were within 15%, satisfying the acceptance criteria for these levels (Supporting Information Table S2).

Process efficiency and absolute recovery of nirmatrelvir in hemolyzed and nonhemolyzed EDTA plasma were consistently around 100% across three different QC levels (QC1 32.7 ng/mL, QC3 1522 ng/mL, and QC4 2440 ng/mL). Process efficiency ranged from 98% to 107% for nonhemolyzed plasma and 96%–98% for hemolyzed plasma, while absolute recovery ranged from 101% to 104% for nonhemolyzed and 101%–111% for hemolyzed plasma. Nirmatrelvir-D9 also showed consistent recovery for both process efficiency and absolute recovery, ranging from 96% to 111% in both plasma types. The matrix factor (MF) for all QC levels in both hemolyzed and nonhemolyzed EDTA plasma was near 1 (range 0.94–0.99) for both nirmatrelvir and nirmatrelvir-D9, indicating minimal matrix effects. Normalized matrix effects (MF-nirmatrelvir/MF-nirmatrelvir-D9) were also close to 1 (range 1.00–1.04), with low variation (Supporting Information Table S3).

The stability of nirmatrelvir at two QC levels, QC1 (32.7 ng/mL) and QC4 (2440 ng/mL), were tested both in hemolyzed and nonhemolyzed EDTA plasma for short-term stability at ambient temperature (22°C), at 4°C–6°C, during five freeze–thaw cycles, reinjection reproducibility (autosampler, 10°C), pretreated samples, and viral deactivation by heat treatment (57°C, 30 min) with and without concomitant medication (ritonavir). Nirmatrelvir did not change significantly and was within ±15% of nominal value in all short-term tests (Table 2). Long-term stability for 1.3 years in −80°C also produced very good result, indicating that nirmatrelvir was stable under these conditions. Even the heat treatment did not negatively impact the analysis.

### 3.2. Clinical Routine Analysis

The developed and validated method was implemented in our high-throughput routine analysis setting and applied in the quantification of patient samples collected in the PLATCOV trial. A total of 458 plasma samples were analyzed, with 52% exceeding the ULOQ, which were reanalyzed using 10-fold dilution with blank EDTA plasma. Most samples above the ULOQ were collected from patients close to dosing events, likely due to high drug concentrations around peak concentrations in patients. The method exhibited robust precision and accuracy, with 131 out of 132 QC samples meeting the acceptance criteria of ±15% deviation across all concentration levels. Although one QC sample failed, the overall average accuracy for all QC levels (QC1 to QC4) remained within the criteria, with values of 101%, 101%, 100%, and 101%, respectively. Additionally, the %CV were below 4%. To assess the reliability and reproducibility of the validated methods, a random selection of patient samples (approximately 8.6% or 19 out of 219 samples within the linear range) was chosen for ISR across the concentration–time profile. These selected samples were reanalyzed in a separate run for ISR evaluation. All 19 reanalyzed samples demonstrated 100% reproducibility, with the percentage differences between the first and second batch ranging from −5.3% to 11.6%. This falls well within the required ±20% range for at least 67% of reanalyzed samples [14].

**Figure 3:**
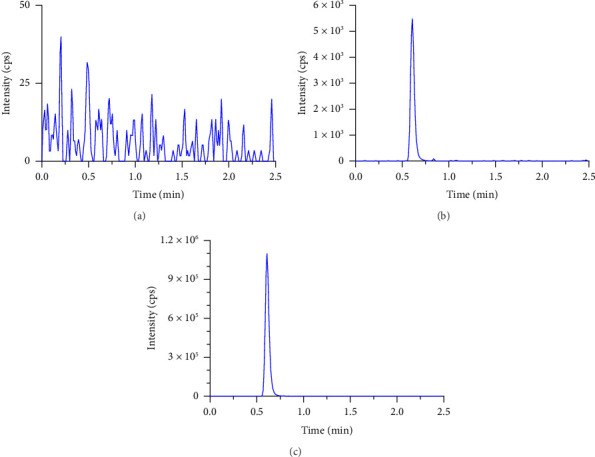
Extracted ion chromatograms of nirmatrelvir during routine sample analysis of patient plasma samples from the PLATCOV trial. Graphs show (a) blank plasma, (b) LLOQ (10.9 ng/mL), and (c) patient sample at 1 h after first dose.

**Figure 4:**
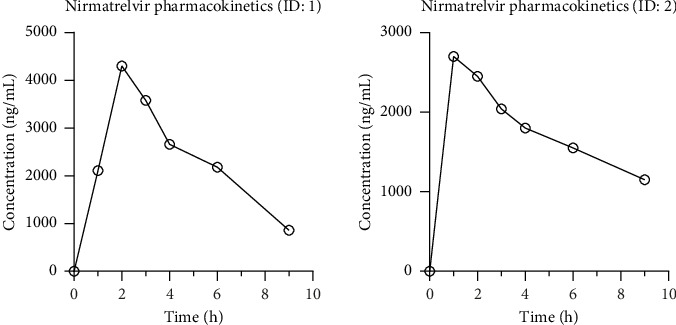
Plasma concentration–time profiles on the first day after an oral dose of nirmatrelvir in two patients.

The results demonstrated adequate separation of nirmatrelvir when analyzing a blank sample (0 ng/mL), a calibrator at LLOQ (10.9 ng/mL), and a patient plasma samples at 1 h after dosing (2110 ng/mL), as shown in Figure 3. Two typical patient plasma concentration–time profiles after an oral dose of nirmatrelvir are shown in Figure 4. Frequent plasma samples were collected between 0 and 12 h postdose, with all concentrations remaining well above the LLOQ (data not shown). These findings are consistent with prior pharmacokinetic studies, indicating that the method can effectively capture the pharmacokinetic properties of nirmatrelvir in patients. The results confirm that the method is suitable for clinical and research use, providing insights into nirmatrelvir's pharmacokinetics and supporting its evaluation in large-scale studies.

## 4. Conclusion

In this study, we present a validated LC-MS/MS method for rapid and precise quantification of nirmatrelvir in human plasma. While the linear range or our developed assay does not cover all of the very high peak concentrations seen in some patient samples, it performed exceptionally well with a 10-fold dilution, ensuring reliable quantification across a broad range of plasma concentrations. Our validation process confirmed that the method meets all required criteria, including specificity, selectivity, linearity, stability, precision, and accuracy, ensuring its reliability for analysis of nirmatrelvir in plasma samples. The use of the Ostro 96-well phospholipid removal plate for sample preparation, simplified the sample extraction process and provided a cleaner plasma sample compared to traditional protein precipitation techniques. Unlike protein precipitation that typically leaves interfering residual proteins and phospholipids behind, the Ostro plate effectively removes phospholipids and reduces the risk of matrix effects during the analysis. This extraction technique was automated using the Freedom Evo100 platform, achieving an automated sample preparation process that enhanced reproducibility and throughput. Furthermore, the total LC-MS/MS analysis run time was reduced to only 2 min per injection, significantly improving efficiency over other methods, which typically require 4–13 min [6–9, 11]. Our method demonstrated to be a high-performing and robust assay also in the face of potential matrix effects, showing no interference from hemolyzed plasma samples, which further validates its reliability under clinical conditions. In summary, our method offers improvements in speed, efficiency, and reproducibility, making it well-suited for large-scale clinical studies and routine quantification of nirmatrelvir in human plasma samples.

## Figures and Tables

**Table 1:** Published methods of nirmatrelvir in human plasma using LC-MS/MS.

Plasma volume (μL)	Extraction method	Column type	Mobile phase	Elution of solvent system	Internal standard	Extraction recovery (%)	Analysis run time (min)	Linear range (ng/mL)	Reference
50	Ostro phospholipid removal plate	Gemini C18 (50 × 2.0 mm, 5 μm)	ACN: 10 mM NH_4_HCO_2_ + 0.5% (v/v) FA (60:40, v/v)	Isocratic	Nirmatrelvir-D9	98–107	2	10.9–3013	Developed method
100	PP	Agilent poroshell 120 SB-C18 (75 × 2.1 mm, 2.7 μm)	ACN: 0.1% (v/v) FA in H_2_O (52:48, v/v)	Isocratic	Selinexo	92–107	3.65	2–5000	[6]
50	PP	Thermo BDS hypersil C18 (100 × 4.6 mm, 2.4 μm)	0.1% (v/v) FA in H_2_O: 0.1% (v/v) FA in MeOH	Gradient	Remdesivir	95–97	7	50–5000	[7]
100	PP	Pursuit XRs ultra diphenyl (100 × 2.0 mm, 2.8 μm)	H_2_O + 0.02% (v/v) FA:ACN	Gradient	Atazanavir-D6	90–106	4	40–4000	[8]
50	PP	Zorbax XDB-C18 (50 × 2.1 mm, 3.5 μm)	16 mM NH_4_HCO_2_ + 0.1% (v/v) FA:ACN	Gradient	Ritonavir-D6	NA	13	10–10000	[9]
100	PP	UPLC BEH C18 (50 × 2.1 mm, 1.7 μm)	NA	Gradient	PF-07818226	NA	NA	10–10000	[10]
200	PP	Zorbax SB-Aq, (150 × 4.6 mm, 5 μm)	0.1% (v/v) FA:ACN	Gradient	Lamivudine	91–102	12	30–10000	[11]

*Note:* ACN: acetonitrile; H_2_O: water; LC-MS/MS: liquid chromatography-tandem mass spectrometry; MeOH: methanol; mL: milliliter; mm: millimeter; mM: millimolar; ng/mL: nanograms per milliliter; NH_4_HCO_2_: ammonium formate; μm: micrometers; μL: microliter.

Abbreviations: FA, formic acid; PP, protein precipitation; v/v, volume/volume.

**Table 2:** The stability of nirmatrelvir in EDTA plasma (hemolyzed and nonhemolyzed) sample and with concomitant medication (ritonavir).

Storage condition	Sample (ng/mL)	Accuracy (%)	CV (%)
*Nonhemolyzed*			
Freeze–thaw (−80°C), 5 cycles	QC1 (32.7)	103	0.45
	QC4 (2440)	96.7	0.42
Ambient temperature (22°C), 24 h	QC1 (32.7)	96.1	2.3
	QC4 (2440)	95.4	4.3
Refrigerator (4°C–6°C), 24 h	QC1 (32.7)	104	4.8
	QC4 (2440)	97.0	4.6
Reinjection reproducibility (autosampler, 10°C), 125 h	QC1 (32.7)	97.7	3.2
	QC4 (2440)	98.5	0.6
^a^Pretreated, ambient temperature (22°C), 4 h	QC1 (32.7)	98.1	1.0
	QC4 (2440)	98.9	1.9
^a^Pretreated, refrigerator (4°C–6°C), 24 h	QC1 (32.7)	97.8	4.6
	QC4 (2440)	98.8	2.7
^b^Deactivated, heat treatment (57°C), 30 min	QC1 (32.7)	101	5.1
	QC4 (2440)	98.8	3.2
Long term (−80°C), 1.3 year	QC1 (32.7)	95.1	1.7
	QC4 (2440)	97.0	3.1

*Hemolyzed*			
Freeze–thaw (−80°C), 5 cycles	QC1 (32.7)	97.2	4.7
	QC4 (2440)	93.6	2.6
Ambient temperature (22°C), 24 h	QC1 (32.7)	92.8	4.2
	QC4 (2440)	96.2	3.9
Refrigerator (4°C–6°C), 24 h	QC1 (32.7)	95.1	5.3
	QC4 (2440)	95.6	2.7
^a^Pretreated, ambient temperature (22°C), 4 h	QC1 (32.7)	95.7	2.4
	QC4 (2440)	103	2.9
^a^Pretreated, refrigerator (4°C–6°C), 24 h	QC1 (32.7)	98.2	3.5
	QC4 (2440)	91.9	7.7
Long term (−80°C), 1.3 year	QC1 (32.7)	94.3	3.3
	QC4 (2440)	94.7	1.9

*Nonhemolyzed + ritonavir*			
Freeze–thaw (−80°C), 5 cycles	QC1 (32.7)	95.4	4.3
	QC4 (2440)	92.8	2.8
Ambient temperature (22°C), 4 h	QC1 (32.7)	98.7	3.9
	QC4 (2440)	92.8	1.4
Long term (−80°C), 1.3 year	QC1 (32.7)	96.3	2.3
	QC4 (2440)	97.9	2.8

^a^Pretreated: plasma samples were precipitated and maintained under specified conditions to assess stability before extraction.

^b^Deactivated, heat treatment (57°C), 30 min: Plasma samples were heat-treated at 57°C for 30 min in a Memmert water bath, with temperature monitored using a calibrated testo 925 thermometer (S/N 34870196/1221).

## Data Availability

The data that support the findings of this study are available from the corresponding author upon reasonable request.

## Supporting Information

The data provided in the Supporting Information, which also support this study, include the following: Table S1. Back-calculated concentrations of nirmatrelvir standards in EDTA plasma; Table S2. Accuracy and precision of nirmatrelvir in EDTA plasma, including both nonhemolyzed and hemolyzed samples; Table S3. Recovery and matrix effect (MF) of nirmatrelvir in EDTA plasma, including both nonhemolyzed and hemolyzed samples; Figure S1. Extracted ion chromatograms of nirmatrelvir at LLOQ (10.9 ng/mL) from (a) six different donors (A–F), including hemolyzed samples in extracted blank EDTA plasma and (b) with concomitant medication of ritonavir (50 ng/mL) and paracetamol (100 ng/mL); Figure S2. Extracted ion chromatogram of nirmatrelvir at LLOQ (10.9 ng/mL), the first extracted blank EDTA plasma sample after five ULOQ injections, and the first blank solution injected after five ULOQ samples; Figure S3. Extracted ion chromatograms of nirmatrelvir at the ULOQ (3013 ng/mL) without internal standard (IS) and of nirmatrelvir-D9 extracted from the ULOQ sample without IS. The chromatograms show no interference between nirmatrelvir at ULOQ and nirmatrelvir-D9, confirming that nirmatrelvir does not contribute to the nirmatrelvir-D9 signal; Figure S4. Extracted ion chromatograms of nirmatrelvir (left panel) and nirmatrelvir-D9 (right panel) from blank EDTA plasma spiked with nirmatrelvir-D9 (300 ng/mL) after extraction, assessing potential interference of the internal standard (IS) with the analyte across four batches.
