# Supplementary material for: LC-MS/MS Method Validation for Quantification of Nirmatrelvir in Human Plasma
Source: Int J Anal Chem. 2025 Nov 17;2025:6625833. doi: 10.1155/ianc/6625833 (PMC12660627; doi:10.1155/ianc/6625833)
Supplement: Supporting Information — Additional supporting information can be found online in the Supporting Information section. [file 6625833.f1.pdf]

Supplementary material

LC-MS/MS method validation for quantifying of nirmatrelvir in human plasma for clinical use

Natpapat Kaewkhao[1], Joel Tarning[1,2,3], Daniel Blessborn[1,2]

[1] Mahidol Oxford Tropical Medicine Research Unit, Faculty of Tropical Medicine, Mahidol University, Bangkok, Thailand.

[2] Centre for Tropical Medicine & Global Health, Nuffield Department of Clinical Medicine, University of Oxford, Oxford, UK.

[3] Infectious Diseases Data Observatory (IDDO), University of Oxford, Oxford, UK.

- **Table S1.** Back-calculated concentrations of nirmatrelvir standards in EDTA plasma.
- **Table S2.** Accuracy and precision of nirmatrelvir in EDTA plasma, including both non-hemolyzed and hemolyzed samples.
- **Table S3.** Recovery and matrix effect (matrix factor) of nirmatrelvir in EDTA plasma, including both non-hemolyzed and hemolyzed samples.
- **Figure S1.** Extracted ion chromatograms of nirmatrelvir at LLOQ (10.9 ng/mL) from (a) six different donors (A–F), including hemolyzed samples in extracted blank EDTA plasma and (b) with concomitant medication of ritonavir (50 ng/mL) and paracetamol (100 ng/mL).
- **Figure S2.** Extracted ion chromatogram of nirmatrelvir at LLOQ (10.9 ng/mL), the first extracted blank EDTA plasma sample after five ULOQ injections, and the first blank solution injected after five ULOQ samples.
- **Figure S3.** Extracted ion chromatograms of nirmatrelvir at the ULOQ (3013 ng/mL) without internal standard (IS) and of nirmatrelvir-D9 extracted from the ULOQ sample without IS. The chromatograms show no interference between nirmatrelvir at ULOQ and nirmatrelvir-D9, confirming that nirmatrelvir does not contribute to the nirmatrelvir-D9 signal.
- **Figure S4.** Extracted ion chromatograms of nirmatrelvir (left panel) and nirmatrelvir-D9 (right panel) from blank EDTA plasma spiked with nirmatrelvir-D9 (300 ng/mL) after extraction, assessing potential interference of the internal standard (IS) with the analyte across four batches.

TABLE S1: Back-Calculated concentrations of nirmatrelvir standards in EDTA plasma.

Sample (ng/mL)	Measurement (ng/mL)	Accuracy (%)	Precision (%CV)
10.9	10.9	100	2.05
54.3	54.6	101	3.30
209	212	101	5.36
628	639	102	3.77
1883	1896	101	2.58
3013	2890	95.9	3.75

%CV: coefficient of variation. Each concentration was analyzed in duplicate in four separate runs.

TABLE S2: Accuracy and precision of nirmatrelvir in EDTA plasma, including both non-hemolyzed and hemolyzed samples.

Sample (ng/mL)	Measurement (ng/mL)	Accuracy (%)	Precision (%CV)	
			Inter-assay	Intra-assay
Non-hemolyzed				
LLOQ (10.9)	10.9	100	13.0	4.1
ULOQ (3,013)	3,007	100	14.1	8.6
Over-curve (6,623)	6,744	102	7.6	4.0
QC1 (32.7)	32.8	100	10.7	6.5
QC2 (450)	450	100	10.8	4.5
QC3 (1,522)	1,501	98.6	14.5	7.3
QC4 (2,440)	2,353	96.4	11.7	3.3
Hemolyzed				
QC1 (32.7)	31.4	96.0	14.4	6.8
QC4 (2,440)	2,262	92.7	8.5	5.6

%CV: coefficient of variation; QC: quality control; LLOQ: lower limit of quantification; ULOQ: upper limit of quantification (n=5).

TABLE S3: Recovery and matrix effect (matrix factor) of nirmatrelvir in EDTA plasma, including both non-hemolyzed and hemolyzed samples.

Sample (ng/mL)	Absolute recovery [% (%CV)]	Extraction recovery [% (%CV)]	[a] Matrix factor	[b] IS-normalized matrix factor
Non-hemolyzed				
QC1 (32.7)	104 (2.1)	97.7 (2.1)	0.947	1.00
QC3 (1,522)	101(3.0)	98.5 (3.0)	0.968	1.00
QC4 (2,440)	104 (4.3)	107 (4.3)	0.992	1.04
SIL for QC1 (300)	102 (2.4)	96.1 (2.4)	0.943	-
SIL for QC3 (300)	101 (3.1)	98.4 (3.1)	0.965	-
SIL for QC4 (300)	110 (5.4)	107 (5.4)	0.950	-
Hemolyzed				
QC1 (32.7)	101 (2.1)	96.6 (5.1)	0.966	1.01
QC3 (1,522)	101 (3.0)	98.1 (0.4)	0.981	0.991
QC4 (2,440)	111 (4.3)	96.3 (1.3)	0.963	0.997
SIL for QC1 (300)	100 (2.4)	95.9 (2.8)	0.959	-
SIL for QC3 (300)	99.4 (3.1)	99.0 (2.3)	0.990	-
SIL for QC4 (300)	111 (5.4)	96.5 (2.0)	0.965	-

%CV: coefficient of variation; QC: quality control; LLOQ: lower limit of quantification; ULOQ: upper limit of quantification.

[a] Matrix factor for each QC level was calculated using six donors (n = 18), while one donor (n = 3) was used per QC level for hemolyzed samples.

[b] IS-normalized matrix factor (MF) = MF analyte/MF IS.

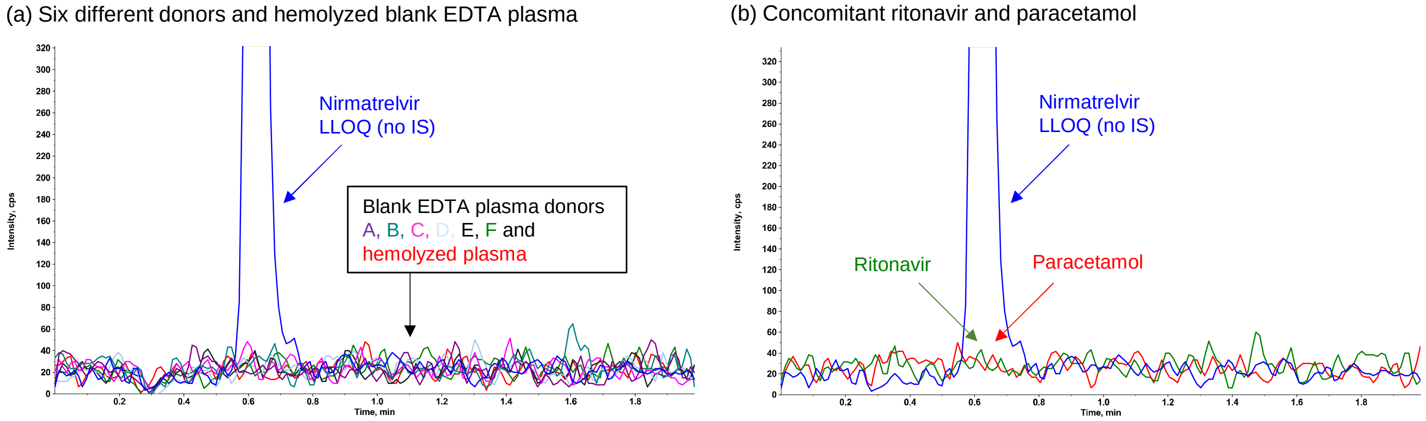

FIGURE S1. Extracted ion chromatograms of nirmatrelvir at LLOQ (10.9 ng/mL) from (a) six different donors (A–F), including hemolyzed samples in extracted blank EDTA plasma and (b) with concomitant medication of ritonavir (50 ng/mL) and paracetamol (100 ng/mL).

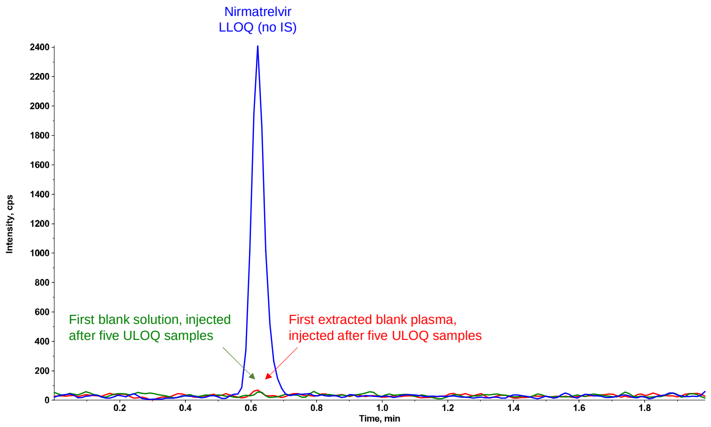

FIGURE S2. Extracted ion chromatogram of nirmatrelvir at LLOQ (10.9 ng/mL), the first extracted blank EDTA plasma sample after five ULOQ injections, and the first blank solution injected after five ULOQ samples.

(a) Nirmatrelvir at ULOQ (no IS)

(b) Nirmatrelvir-D9 extracted from nirmatrelvir at ULOQ

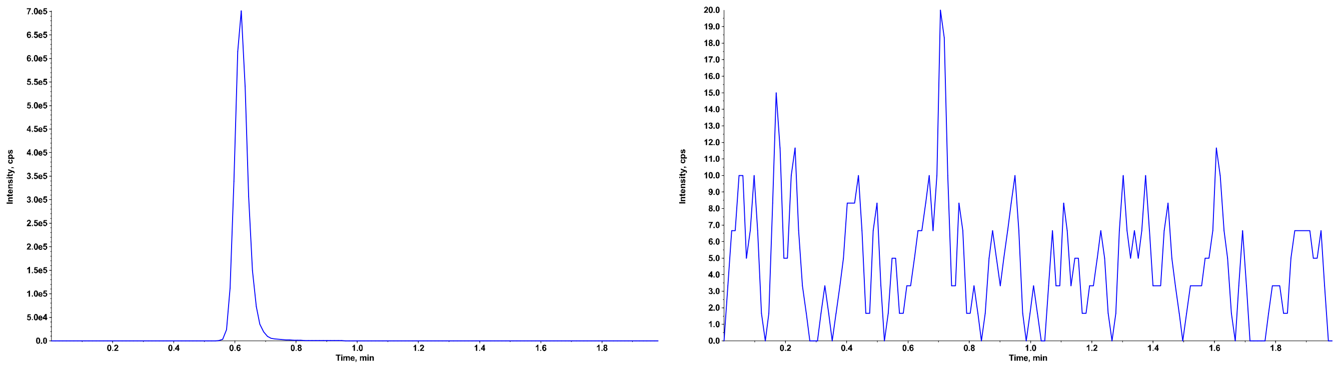

FIGURE S3. Extracted ion chromatograms of nirmatrelvir at the ULOQ (3013 ng/mL) without internal standard (IS) and of nirmatrelvir-D9 extracted from the ULOQ sample without IS. The chromatograms show no interference between nirmatrelvir at ULOQ and nirmatrelvir-D9, confirming that nirmatrelvir does not contribute to the nirmatrelvir-D9 signal.

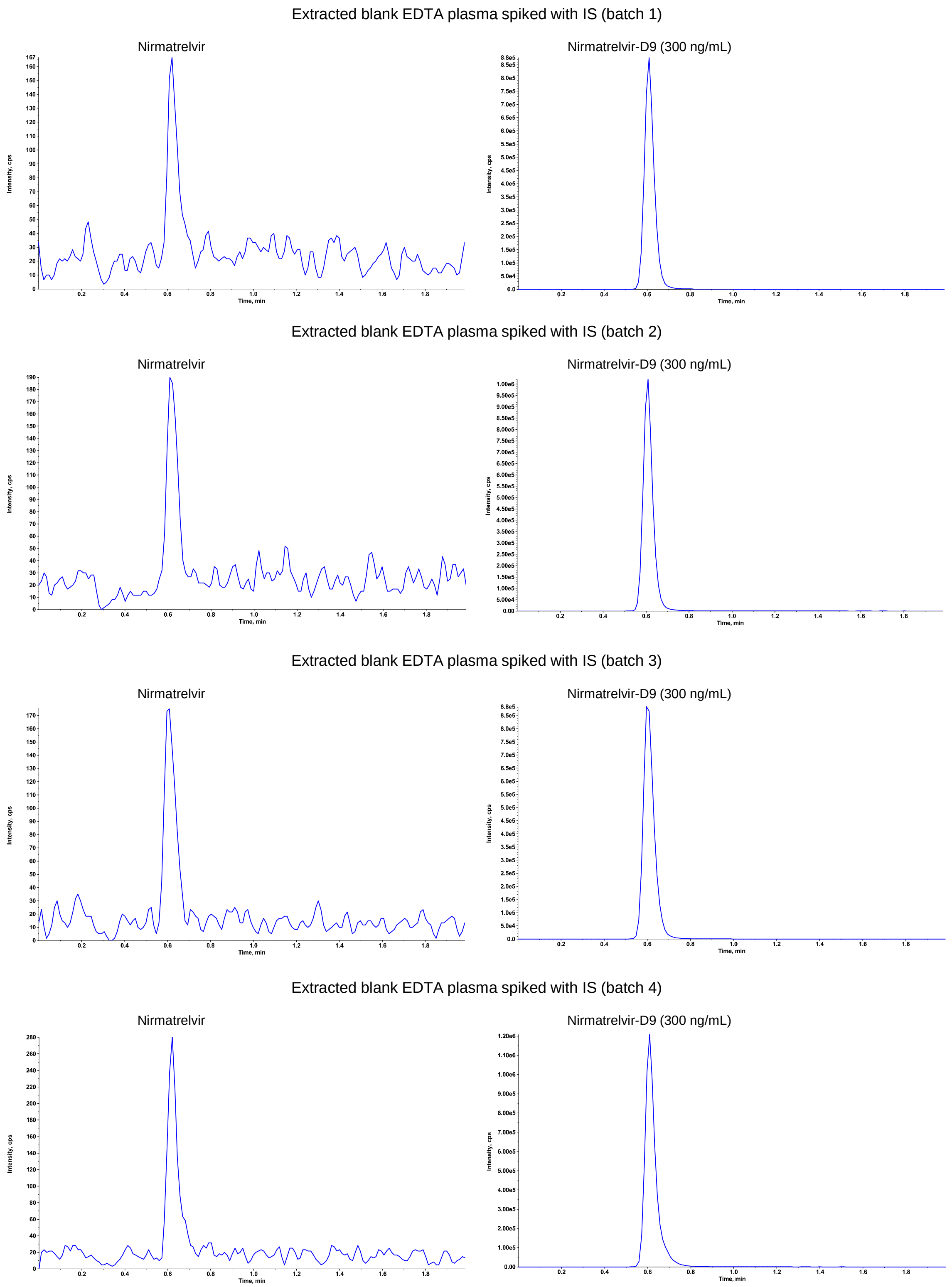

FIGURE S4. Extracted ion chromatograms of nirmatrelvir (left panel) and nirmatrelvir-D9 (right panel) from blank EDTA plasma spiked with nirmatrelvir-D9 (300 ng/mL) after extraction, assessing potential interference of the internal standard (IS) with the analyte across four batches.
